# Supplementary material for: Iterative improvement in the automatic modular design of robot swarms
Source: PeerJ Comput Sci. 2020 Dec 7;6:e322. doi: 10.7717/peerj-cs.322 (PMC7924708; doi:10.7717/peerj-cs.322)
Supplement: Supplemental Information 3 [file peerj-cs-06-322-s003.zip › argos3/doc/api/standalone/a00441_source.html]

ARGoS: plugins/robots/foot-bot/control\_interface/ci\_footbot\_encoder\_sensor.h Source File


- Main Page
- Related Pages
- Namespaces
- Classes
- Files

- File List
- File Members

# plugins/robots/foot-bot/control\_interface/ci\_footbot\_encoder\_sensor.h

Go to the documentation of this file.

```
00001 
00016 #ifndef CCI_FOOTBOT_ENCODER_SENSOR_H
00017 #define CCI_FOOTBOT_ENCODER_SENSOR_H
00018 
00019 /* To avoid dependency problems when including */
00020 namespace argos {
00021    class CCI_FootBotEncoderSensor;
00022 }
00023 
00024 #include <argos3/core/control_interface/ci_sensor.h>
00025 
00026 namespace argos {
00027 
00028    class CCI_FootBotEncoderSensor : public CCI_Sensor {
00029 
00030    public:
00031 
00039       struct SReading {
00040          Real CoveredDistanceLeftWheel;
00041          Real CoveredDistanceRightWheel;
00042          Real WheelAxisLength;
00043          
00044          SReading() :
00045             CoveredDistanceLeftWheel(0.0f),
00046             CoveredDistanceRightWheel(0.0f),
00047             WheelAxisLength(0.0f) {}
00048          
00049          SReading(Real f_covered_distance_left_wheel,
00050                   Real f_covered_distance_right_wheel,
00051                   Real f_wheel_axis_length) :
00052             CoveredDistanceLeftWheel(f_covered_distance_left_wheel),
00053             CoveredDistanceRightWheel(f_covered_distance_right_wheel),
00054             WheelAxisLength(f_wheel_axis_length) {
00055          }
00056 
00057       };
00058 
00062       virtual ~CCI_FootBotEncoderSensor() {}
00063 
00068       const SReading& GetReading() const;
00069 
00070 #ifdef ARGOS_WITH_LUA
00071       virtual void CreateLuaState(lua_State* pt_lua_state);
00072 
00073       virtual void ReadingsToLuaState(lua_State* pt_lua_state);
00074 #endif
00075 
00076    protected:
00077 
00078       SReading m_sReading;
00079    };
00080 
00081 }
00082 
00083 #endif
```

---

Generated on 10 Jul 2018 for ARGoS by 
 1.6.1
